# Racial disparities in cardiovascular manifestations among patients with sickle cell trait: analysis of national inpatient sample data (2016–2020)

**DOI:** 10.1007/s00277-025-06306-0

**Published:** 2025-03-20

**Authors:** Praneeth Reddy Keesari, Charan Thej Reddy Vegivinti, Medha Rajamanuri, Ahmad Mustafa, Ngowari Pokima, Lauren Haley White, Hasnain Chaudhry, Chamberline E Ozigbu, Khairul A Siddiqi, Suzanne El-Sayegh, Meekoo Dhar, Ugochi Olivia Ogu

**Affiliations:** 1 Department of Internal Medicine, Staten Island University Hospital, Staten Island, NY USA; 2 Division of Hematology Oncology, MD Anderson Cancer Center, Houston, TX USA; 3 Department of Internal Medicine, Southern Illinois University, Springfield, IL USA; 4 Department of Cardiovascular Medicine, Staten Island University Hospital, Staten Island, NY USA; 5 Department of Medicine, Ross University School of Medicine, Two Mile Hill St. Michael, Bridgetown, Barbados; 6 Department of Health Services Policy and Management, University of South Carolina, Columbia, SC USA; 7 Community and Behavioral Health, Washington State University Elson S. Floyd College of Medicine, Spokane, WA USA; 8 Department of Hematology/Oncology, Staten Island University Hospital, Staten Island, NY USA; 9 Division of Hematology, University of Tennessee Health Science Center, Memphis, TN USA; 10 Center for Sickle Cell Disease, University of Tennessee Health Science Center, Memphis, TN USA

**Keywords:** Sickle cell trait, Cardiovascular, Racial disparity, African American

## Abstract

Sickle cell trait (SCT) is a condition that affects up to 3 million individuals and 10% of African Americans (AA) in the United States (U.S.). Although often believed to be benign, the true burden of the condition remains understudied. Data is also lacking regarding the racial disparities in SCT and if African American individuals with SCT are at increased cardiovascular risk compared to individuals of other races. Using the National Inpatient Sample (NIS) database, we investigated cardiovascular outcomes in AA individuals with SCT compared to other racial groups. Our study reported statistically significant higher odds of acute heart failure in AA individuals with SCT (Adjusted OR: 1.39 (*p* < 0.01, 95% CI: 1.13–1.71). Further studies investigating the racial disparities and cardiovascular risk among individuals with SCT are warranted to measure the causal effects.

## Introduction

Sickle cell disease (SCD) is easily classified as one of the most common monogenetic diseases globally, with its carrier state, sickle cell trait (SCT) affecting over 300 million people globally [1]. While the severe complications of SCD are well-known, sickle cell trait (SCT) is often considered benign. Sickle cell trait has a protective factor for Malaria and is regularly found in patients who originate from origins where malaria is endemic. Because of migration flows, the prevalence of SCT in the U.S. is around 3 million people. SCT effects 7–9% of the African American population and 0.2% of the Caucasian population in the United States [1].

HbS results from a single point mutation on the beta (β) subunit of hemoglobin chains. Subsequently, adenine replaces thymine, changing glutamic acid to valine. This creates an abnormal hemoglobin S (HbS), which when inherited in a homozygous form or with other abnormal beta globin variants or beta-thalassemia, polymerizes under low oxygen tension and causes erythrocytes to lose their characteristic shape, resulting in a sickled erythrocyte. In SCD, these sickled cells have potential to cause dire health emergencies especially in setting of high altitudes or strenuous exertion [1]. Although SCT carriers do not have the usual manifestations of SCD, carriers have up to 40% hemoglobin S. Unlike SCD, individuals with SCT typically live a normal life and are asymptomatic. Rare complications especially under extreme conditions and high altitudes include rhabdomyolysis and splenic infarction.

It is hypothesized that activated coagulation system, arteriopathy, and cardiac remodeling plays a part in the pathophysiology of increased cardiovascular pathology in patients with SCT [2–4]. Recent evidence suggests that sickle cell trait may be associated with more than vaso-occlusive related complications at high altitudes [5, 6]. Michelon et al. in a meta-analysis reported an elevated risk of hospitalization and renal injury in SCT individuals with COVID-19, emphasizing need for further research into SCT associated risks across broader health conditions [7]. High quality studies are lacking to confirm or exclude elevated cardiovascular risk in patients with SCT, particularly of AA origin. Health disparities in cardiovascular disease between African Americans and non-Hispanic whites are increasingly reported [8]. Given the emerging view of race as a social construct and the lack of existing literature, we examined the differences in adverse cardiovascular outcomes between African American and non-African American carriers of SCT.

## Methods

### Data source

A retrospective analysis was conducted using the National Inpatient Sample (NIS) data from 2016 to 2020 [9]. We were not required to obtain Institutional Review Board permission, as this study utilized publicly available de-identified data.

### Inclusion criteria

We included adult inpatients ≥ 18 years of age with a principal diagnosis of SCT (*N* = 272,035) by identifying the International Classification of Diseases (ICD)-10 codes in the diagnoses field DX1 in the NIS. From the study population, we then identified individuals with major adverse cardiovascular events.

### Variables of interest

Demographic variables evaluated in this study were age, sex, race, median household income, and insurance coverage. To measure the differences in outcomes in SCT inpatients by race, the following primary outcomes and major adverse cardiovascular events were included: acute myocardial infarction (MI), cardiac arrest, acute pulmonary edema, valvular disease, pericarditis, acute heart failure, pulmonary hypertension, tachyarrhythmia, conduction disorders, stroke, acute pulmonary embolism (PE), acute deep venous thrombosis (DVT). Secondary outcomes of interest included in-hospital mortality, mean hospital charges, and length of stay (LOS). The LOS refers to the number of nights the patient remained in the hospital for the principal diagnosis.

### Statistical analyses

We used chi-square test for univariate analysis and ANOVA for multivariate regression analysis to measure the differences in outcomes for African American and other race carriers of SCT for multiple adverse cardiac events. We used a multivariable logistic regression model to evaluate the risk of primary outcomes and secondary outcomes in African American SCT carriers versus patients of other races with SCT. The model was adjusted for age, gender, income quartile, Charlson comorbidity index, hospital region, and hospital size. A p value < 0.05 was used to determine the statistical significance of the test results. All statistical analyses were conducted using Stata BE/18.0.

## Results

We identified a total of 272,035 SCT admissions between 2016 and 2020, of which 233,210 individuals were of AA race and 38,825 were of other races. Mean age was 36 years and 30 years in AA and non-AA groups respectively. 80% of individuals in both groups (186,568 in AA vs. 31060 in non-AA) were females. Comorbidities, income quartiles, and insurance coverages are reported in Table 1.

Statistical analysis of the comparison of outcomes between AA and non-AA individuals with SCT showed that acute heart failure was associated with higher unadjusted (OR 2.31, *p* < 0.01 95% CI 1.90–2.83) and adjusted odds (OR 1.39, *p* < 0.01 95% CI 1.13–1.71). Acute MI (Unadjusted OR: 1.69 (*p* < 0.01, 95% CI: 1.27–2.25), valvular disease (Unadjusted OR: 1.33 (*p* < 0.01, 95% CI: 1.07–1.67)), pulmonary hypertension (Unadjusted OR: 1.71 (*p* < 0.01, 95% CI: 1.38–2.12)), tachyarrhythmia (Unadjusted OR 1.46 (*p* < 0.01, CI: 1.30–1.62)), and acute DVT (Unadjusted OR 1.52 (*p* < 0.01, CI: 1.18–1.94)), although had statistically significant unadjusted odds in AA individuals with SCT compared to non-AA individuals with SCT, did not show statistically significant higher adjusted odds (Table 2). Cardiac arrest, acute pulmonary edema, pericarditis, conduction disorders, stroke (including TIA), acute PE, in-hospital mortality were not found to have higher unadjusted or adjusted odds in AA individuals with SCT as compared to non-AA individuals with SCT (Table 2). Mean LOS was not different among AA individuals with SCT (5.02 days) compared to non-AA individuals with SCT (5.06 days). Mean hospital charges incurred by AA individuals with SCT were slightly lower than non-AA individuals with SCT ($48,815 vs. $53,714) (Table 2).

## Discussion

To our knowledge this is the first study analyzing cardiovascular risk in African American individuals with SCT, compared to individuals of other races with sickle cell trait. Our study reported 39% higher odds of acute heart failure in AA individuals with SCT than non-AA individuals with SCT. In our analysis, acute myocardial infarction, valvular diseases, pulmonary hypertension, tachyarrhythmia and acute DVT, although showed higher odds in AA individuals with sickle cell trait initially, did not achieve statistical significance after adjusting. Hyacinth et al. found no increased risk of coronary heart disease and myocardial infarction in AA individuals with SCT, compared to AA individuals without SCD, after adjustment for confounders based on data from 5 population-based cohorts [4]. It should however be noted that the overall study groups had significantly higher women (70% vs. 30%) population than men and analysis within the 5 population cohorts showed higher CHD events in patients with SCT in the Multi-Ethnic Study of Atherosclerosis group population that had higher men (46%). Another analysis of patients with SCT and CKD showed higher coronary artery disease in male patients with SCT but not among females with SCT [10]. Ajayi et al. also reported higher rates of retinopathy in AA males with diabetes and SCT but not in the female counterpart [11]. Our study also consisted of higher female individuals in both groups (80% in both AA and non-AA SCT groups). Further studies with adequate power will be needed to perform sex stratification and clarify the elevated coronary artery disease among male patients with SCT. Although data is lacking for valvular disease in sickle cell trait patients, one study based on 4 population-based cohorts found no significant higher tricuspid valve abnormalities in AA SCT population compared to non-SCT population [12]. Pulmonary hypertension is an established factor for SCD [13]. However, its risk in patients with SCT is unknown, and our study showed no elevated risk in AA SCT population after adjusting for confounders. Studies have reported abnormal coagulation profiles in individuals with SCT including elevated d-dimer [3]. A population-based cohort study of 268 AA individuals with SCT, Folsom et al. reported 1.5 times higher odds of overall venous thromboembolism and 2 times higher odds of pulmonary embolism in AA individuals with SCT compared to other AA individuals but interestingly no elevated DVT risk in this population. In another case-control study Austin et al. found elevated overall VTE and PE risk but no significant risk of DVT in AA individuals with SCT [14]. Both studies could not explain the lack of elevated risk for DVT in AA individuals with SCT, although possible misclassification was reported and controls in Austin et al. study were enrolled from clinic visits and the individuals with SCT were patients admitted to hospital. Studies have also hypothesized this to a possible higher risk of embolization of DVT in patients with SCT [15].

Our study found higher risk of acute heart failure in AA individuals with SCT compared to non-AA individuals with SCT, even after adjusting for confounders (Adjusted OR: 1.39 (*p* < 0.01, 95% CI: 1.13–1.71)). Bello et al. investigated the risk of heart failure in 15,364 AA patients (1,211 patients had SCT) and reported no increased risk of heart failure in AA individuals with SCT compared to AA individuals without SCT [12]. Majority of this cohort was females (around 70%) which could have affected the results. Another analysis of around 800 individuals with SCT showed higher odds of left ventricular hypertrophy in younger AA patients with SCT [2]. Further high-quality studies evaluating risk of heart failure in patients with SCT are lacking.

Risk of stroke was not significantly higher in AA patients with SCT compared to other races (Unadjusted OR: 1.21 (*p* = 0.150, 95% CI: 0.93–1.58)), including after adjusting for confounders. Prior studies showed mixed evidence regarding the risk of stroke in patients with SCT. A population-based cohort study of relatively small sample (233 individuals with SCT) reported a hazard ratio of 1.4 in AA patients with SCT compared to AA patients without SCT [16], while another meta-analysis including 1520 individuals with SCT reported no higher risk of stroke from SCT, suggesting need to sort for other possible causes in AA patients with SCT and stroke [17].

Tachyarrhythmias although showed higher unadjusted odds (Unadjusted OR: 1.46 (*p* < 0.01, 95% CI: 1.30–1.62)) did not remain statistically significant after adjusting for confounders. Douce et al. in an analysis of around 800 AA individuals with SCT suggested that Atrial fibrillation rates were higher in AA individuals with SCT despite adjusting for hypertension and chronic kidney disease status [18]. There is otherwise limited data analyzing conduction abnormalities in patients with SCT.

Overall, there are several inconsistencies regarding the cardiovascular risk in AA individuals with SCT. Our study suggested that AA individuals with SCT may be at higher risk of heart failure than individuals with SCT of other races but failed to reveal otherwise elevated cardiovascular risk. The lack of significance of several cardiovascular outcomes on adjusting suggests how certain factors like age, gender, race, socio-economic status and other comorbidities mediate health disparities and their complex interplay in this study of association of adverse cardiovascular outcomes in AA individuals with SCT. The persistence of higher odds of acute heart failure would require clinicians managing patients with SCT to be aware of this association and aggressively manage other cardiovascular comorbidities such as smoking, obesity, high blood pressure, hyperlipidemia, etc., in this population. Further large-scale studies will be necessary to evaluate the association between adverse cardiovascular outcomes and SCT in AA individuals. If additional associations are found, the role of medications as primary prevention for cardiovascular diseases should be investigated.

### Strengths, limitations, and future perspectives

Main strength of this study is the inclusion of a large and diverse nationally representative study population ensuring better generalizability to the US population. Multiple cardiovascular outcomes have been analyzed in this study ensuring a comprehensive approach. The study also addresses a significant gap in the literature in comparing racial disparities in cardiovascular outcomes in patients with SCT. The use of adjusted analysis further strengthened the validity of the results. While further large-scale studies using individual level data will be needed to confirm the results of this analysis, we believe our study is a valuable addition to the existing literature. The study may be limited by difference in the number of patients in AA and other race cohorts at baseline, retrospective nature of the NIS database, underreporting, coding error, misclassification, and lack of matching. Although the study has adjusted for age, gender, income quartile, Charlson comorbidity index, hospital region, and hospital size, possible confounding from other cardiovascular comorbidities and genetic factors cannot be excluded given limited clinical data available. Further large-scale prospective studies with more clinical and genetic data would be required to validate current results and establish causality. Inclusion of non-hospitalized patients, lifestyle factors, and other comorbidities data will improve generalizability and provide more comprehensive insight into racial disparities in individuals with SCT.

## Conclusion

Our study reported 39% higher odds of acute heart failure in AA individuals with SCT than non-AA individuals with SCT. Healthcare providers caring for individuals with SCT should be aware of this association and aggressively manage comorbid conditions like hypertension, diabetes, smoking, obesity, hyperlipidemia etc. in this population. Future large-scale prospective studies investigating the cardiovascular risk in individuals with SCT, particularly AA population will be needed to validate the results of our study and understand underlying pathophysiologic mechanisms involved.

**Table 1:** Baseline characteristics of individuals with SCT

Baseline characteristic	AA	Non-AA
Age (mean years)	36	30
Female	186,568(80%)	31,060(80%)
Male	46,642(20%)	7,765(20%)
Charlson comorbidity index		
0	125,933(54%)	26,401(68%)
1	41,978(18%)	6,600(17%)
2	186,57(8%)	2,329(6%)
3	46,642(20%)	3,494(9%)
Median household income Quartiles		
1	118,937(51%)	15,530(40%)
2	51,306(22%)	8930(23%)
3	39,646(17%)	8153(21%)
4	23,321(10%)	6212(16%)
Insurance coverage		
Medicare	41,978(18%)	3882(10%)
Medicaid	144,273(49%)	20,965(54%)
Private insurance	67,631(29%)	12,036(31%)
Self-pay	9328(4%)	1941(5%)

AA- African American; SCT– Sickle cell trait

**Table 2:** Cardiovascular outcomes in AA individuals with SCT compared to other races

Outcome	AA	Other race	Unadjusted odds ratio (*p* value) (95% CI)	Adjusted odds ratio (*p* value) (95% CI)^a^
Acute MI	2379	39	1.69 (< 0.05) (1.27–2.25)	1.03 (0.83) (0.77–1.39)
Cardiac arrest	840	19	1.21 (0.34) (0.81–1.81)	0.80 (0.31) (0.52–1.23)
Acute Pulmonary edema	443	8	1.60 (0.13) (0.86–2.98)	1.02 (0.94) (0.53–1.96)
Valvular disease	3148	66	1.33 (< 0.05) (1.07–1.67)	0.90 (0.37) (0.71–1.13)
Pericarditis	140	2^b^	1.89 (0.29) (0.57–6.18)	2.1 (0.26) (0.57–7.81)
Acute heart failure	6,553	82	2.31 (*p* < 0.05) (1.90–2.83)	1.39 (< 0.05) (1.13–1.71)
Pulmonary Hypertension	5,014	82	1.71 (< 0.05) (1.38–2.12)	1.09 (0.41) (0.88–1.3)
Tachyarrhythmia	15,835	311	1.46 (< 0.05) (1.30–1.62)	1.10 (0.08) (0.98–1.23)
Conduction disorders	2,029	47	1.16 (0.238) (0.90–1.50)	0.86 (0.261) (0.66–1.12)
Stroke (Including TIA)	2,006	47	1.21 (0.150) (0.93–1.58)	0.78 (0.088) (0.59–1.04)
Acute PE	2,565	54	1.32 (*p* < 0.05) (1.03–1.67)	1.14 (0.30) (0.89–1.46)
Acute DVT	2,729	50	1.52 (< 0.05) (1.18–1.94)	1.14 (0.296) (0.89–1.47)
In-Hospital Mortality	1,469	35	1.17 (0.3) (0.87–1.58)	0.76 (0.087) (0.56–1.04)
LOS Mean (95% CI)	5.02 (4.92–5.11)	5.06(4.84–5.27)		
Total charge, Mean (95% CI)	48,815 (47489–50141)	53,714 (50310–57118)		

AA- African American; MI: Myocardial Infarction; TIA: Transient Ischemic attack; PE: Pulmonary embolism; DVT:

Deep venous thrombosis; LOS: Length of stay; Total charge in USD; a - The model was adjusted for age, gender, income quartile, Charlson comorbidity index, hospital region, and hospital size; b– To be noted that *N* < 30 would not be a valid estimate in a national level data

## Data Availability

The data that support the findings of this study are available from the corresponding author upon reasonable request.
